# Case of Induration of the Cellular Membrane in a Child

**Published:** 1827-02

**Authors:** Mac Andrew

**Affiliations:** the South London Dispensary.


### INDURATION OF THE CELLULAR MEMBRANE.

*Case of Induration of the Cellular Membrane in a Child.* Treated by Dr. Mac Andrew, at the South London Dispensary.

George Horwich, eighteen months old, admitted a patient at the South London Dispensary, October 16th, 1826.

His mother states that, about two months ago, he was observed to be feverish and restless, and soon afterwards became affected with a looseness of the bowels, and a swelling of the lower extremities; which complaints have never left him since. He has taken powders, with the nature of which she is unacquainted, and has been cupped behind the ears. When examined to-day, the feet, legs, and thighs are found to be much enlarged, in consequence of a diffused swelling that renders the skin very tense. The swelling has a wax-like appearance, and is nearly colourless, excepting for about two inches in the middle of the left leg, where it has a livid colour. Some degree of livor is also observable on the thighs. The swelling does not pit, although firmly pressed by the fingers; nor does the pressure appear to occasion any pain, except on the discoloured part of the left leg. All the affected parts feel cold to the touch. The penis is much distended, but not livid. The abdomen is neither swollen nor hard; the rest of the body is emaciated. He has nearly twelve stools daily: they are of a yellow colour, sometimes greenish; they are very liquid, and occasionally squirted out to a considerable distance. He appears to be pained before having an evacuation. He makes water freely. He takes the breast readily enough, but refuses other nourishment.

Pulv. Cretæ comp. gr. v. ter die.—Liquor. Ammon. Acetat f. ʒiij.; Spirit. Rectificat. f. ℥ j.; Aquæ f. ℥ viij. M. fiat lotio continuo admovenda.

18th.—The swelling of the thighs is very much diminished, and that of the legs, feet, and penis, is also considerably lessened. That part of the left leg which was before of a livid colour, has now acquired a dark red colour, as if fresh blood had been effused from a bruise. The feet and legs are still cold, and the lotion has been applied tepid. Bowels not so frequently opened, and stools less watery. He is very thirsty, and has taken beef-tea freely. Tongue clean. He looks very pale and weak.

Continue.

19th.—Little change in the swelling. Diarrhœa continues: the stools are not quite so liquid; they are of a yellow colour, and in one of them a little blood has been observed. Does not take so much beef-tea.

Contin$^{r}$ pulveres.—Mucilag. Amyli. f. ℥ ss.; Tinct. Opii m. vj. pro enemate.

20th.—Enema returned immediately: he has only had one stool since. Passed the night quietly enough, but towards morning he became apparently insensible, and was observed to make a noise in breathing. Face pale; breathing laborious, and attended with a mucous rattle. Pulse at the wrist still perceptible.

To take from time to time a little gruel mixed with brandy.

Died at ten next morning.

The body was examined twenty-eight hours after death.

*Chest:* Some frothy mucous fluid in the trachea and bronchi, although the inner membrane shows no redness or other sign of inflammation. The lungs are healthy throughout; the posterior part of the inferior lobes being of a darker colour than the rest, merely on account of the position of the body. The heart is of the natural size, and contains polypi in the right auricle and ventricle.

*Abdomen:* Intestines distended with flatus. Stomach contracted, and contains a little gruel; its mucous membrane is corrugated, and quite pale, excepting a minute point of a red colour in the great sac. Duodenum healthy, containing bile of a rich yellow colour. The jejunum is of a red colour externally, for an inch or two in different parts: these portions of intestine are contracted, and their inner membrane is red, but not ulcerated. The same appearance is observed in several parts of the ileum. About half way between the duodenum and cœcum, the upper part of the intestine had slipped into the lower for an inch and a half: the intussuscepted part, although much contracted, was neither red nor inflamed. The caput cœcum coli was filled with healthy-looking fæces, but its inner membrane was much reddened. The colon on the left side was much contracted, and at the sigmoid flexure the redness of the mucous membrane was very marked.

*Extremities:* The cellular membrane of the left leg is much thickened. It is more than a quarter of an inch in depth; it is of a light red colour; is very dense, and does not yield on pressure; it has a distinct granular appearance, and is not unlike a portion of hepatised lung. Immediately below this indurated membrane, there is a layer of gelatinous looking substance, of about two lines in thickness, which, when cut into, allows a thin fluid to escape. This change of structure is observed over the whole left leg, and also prevails to a certain extent in the thigh and leg of the right side. A puncture made in the foot allowed a few drops of fluid to escape, on firm pressure being applied. The muscles of the leg were perfectly healthy.

*Head* not examined.

There is nothing very remarkable in the diarrhœa which carried off this child: it was evidently caused by inflammation in different parts of the intestinal tube; and although, in all probability, this was of two months' duration, it had not excited ulceration. The affection of the extremities, however, is an uncommon disease, and the cases of it described in this country are but few. Excepting the effects of severe external injury, the most common causes of a general swelling of the limbs are erysipelas and anasarca. These two affections are very different in their nature; they scarcely agree in any point but the mere swelling: in the one case, accompanied with burning heat, vivid redness, and acute fever; in the other, characterised by a want of colour and of pain, a scanty flow of urine, and a pitting upon pressure that is very remarkable.

There are three diseases that appear to occupy a place between these two extremes, and in which their symptoms are more or less blended together,—the phlegmasia dolens, which is peculiar to women in child-bed; the Barbadoes leg, or elephantiasis of some authors, which is almost confined to warm climates; and a general induration of the subcutaneous cellular web, that has chiefly been observed in infants,—the "skin-bound" of Dr. Underwood, the "endurcissement du tissu cellulaire" of the French pathologists.

I believe the case of Horwich to be an example of this last affection; at least it approaches nearer to it than to any other disease that I have ever heard of. With regard to the livid colour which was observed in part of the leg in this case, it is by no means a constant symptom of the disease. Dr. Denman never mentions it, but says that the colour of the skin is always yellowish-white; and Dr. Underwood agrees with this description.* In the cases observed at Paris, however, Andry and Auvity frequently met with a bluish colour of the affected parts. M. Dard likewise remarked it in the case that he has related.† In the present instance, it had very much the look of a part that had been much bruised.

The coldness of the affected parts is, perhaps, the most singular feature in the complaint. It was so evident and so unexpected in the first case ever related, that the midwife declared that the child felt like a lump of ice, in coming into the world. It is noticed by all who write on the subject; and the patients are frequently described as receiving the impression of external heat like any inanimate body. They are warmed at the fire like a piece of wood, and become cold again in spite of the use of warm flannels. It does not, however, appear that the sensibility of the part is destroyed. Patrizia Galiera retained the power of feeling, although the skin had lost its natural warmth.‡ In the case of Horwich, there was

* Underwood's Diseases of Children.

‡ Case extracted from the Italian of Carlo Crusio, and related in the Philosophical Transactions, vol. xlviii.

pain at a particular part of the swelling, but this does not appear to have been a common occurrence.

The hardness of the intumescence, and the firmness with which it resists pressure, show the difference between this complaint and anasarca. In the case related by Uzembezius, no impression was made on the cheeks by the finger; the parts affected resembled flesh hardened in the smoke. In the Neapolitan girl, the flesh felt hard to the touch, like wood or a dry hide. Dr. Denman found the skin and flesh hard and resisting, and the cellular membrane fixed in such a manner that it would not slide over the muscles. Similar remarks are made by other authors.

The parts to which the disease extends vary considerably: in some the lower limbs only are affected, as in Horwich; in others, the lower part of the abdomen, the scrotum, the upper extremities; the neck and the face are also involved: hence difficulty of opening the mouth, and inability to bend the neck, to turn the head, or to use the limbs.

The subjects of this disease are generally children not more than ten days old. M. Dard's patient was a year old; Horwich one year and a half; whilst Crusio's patient is the only adult who has evidently laboured under the disease, and whose case has been related.

Mr. White, when speaking of phlegmasia dolens, says that he has seen another disorder of the same kind, without pain or fever, and of several years' standing. Hewson also saw two such cases; and it is not improbable, from the brief descriptions given, that they may have been instances of this complaint.*

The appearances on dissection are not uniform. Underwood never discovered any extravasation in the cellular membrane after death. Andry and Auvity always found an incision followed by an abundant flow of serosity, of a deep yellow colour, and of an albuminous nature, coagulating by heat. The cellular membrane was granulated, compact, and dry. In M. Dard's case, the diseased part offered much resistance to the knife; no serosity exuded from it, and the cellular membrane looked like that of a recently killed hog. When Galiera was bled, the resistance offered to the incision was so great that the lancet yielded and was bent. Breschet generally found the foramen ovale open in the subjects of the disease; but I am not aware that this coincidence has been remarked by other authors.

The cause of this affection is far from being well ascer-

* Inquiry into the Nature, &c. of a Swelling in Lying-in Women.

tained. At Paris, it generally attacked children who had been exposed to the action of cold from improper clothing. Underwood attributed it to the unhealthy air of crowded hospitals, or of the habitations of the poor; and is disposed to look upon it as epidemic at certain seasons. He almost always found it connected with diarrhœa advanced to the last stages; and in this case the same connexion was observed. The French cases have often been complicated with spasmodic affections, approaching in their nature to tetanus: frequently, however, there was no complication, and the patients recovered. In the majority of cases the complaint has proved fatal, perhaps more on account of the other diseases under which the patients laboured at the same time, than from the effects of the disorder itself. The danger diminishes as the age advances.

The treatment recommended by Underwood consisted in a strict attention to the state of the bowels, and the administration of ammonia as a stimulus; warm bathing and warm clothing being the only topical means employed. Andry used a decoction of sage, or other aromatic herbs, as a local application; and he was frequently successful. Dard combined these with the use of purgatives. The Neapolitan girl used baths of milk and water, decoction of woods, vapour bath, and pure mercury; and she recovered.

The most remarkable circumstance in the case of Horwich is the rapid diminution of the swelling which took place in two days, although it had been two months in forming. I know not to what this activity of absorption can be attributed, unless to the effects of the lotion employed; but I certainly did not expect that it would have proved so efficacious. Of course, it was only the thinner fluids that were absorbed; for we cannot suppose that the compact tissue, observed after death, was at all affected in so short a time.

Upon the whole, the most rational plan of treatment would appear to be, to direct the chief attention to the more serious disease with which this affection is generally complicated; and, when this has been removed, to have recourse to the usual means of promoting the absorption of effused fluids, or the resolution of chronic indurations,—namely, friction, pressure, and the internal exhibition of mercury.

161, *Great Surrey-street;*
*December*, 1826.

